# Sleep Slow‐Wave and Spindle Alterations in Young Smokers Correlated With the Severity of Cigarette Exposure

**DOI:** 10.1111/adb.70149

**Published:** 2026-04-05

**Authors:** Shuailin Ding, Ting Xue, Fang Dong, Yongxin Cheng, Yuxin Ma, Juan Wang, Dahua Yu, Kai Yuan

**Affiliations:** ^1^ School of Digital and Intelligent Industry Inner Mongolia University of Science and Technology Baotou Inner Mongolia China; ^2^ School of Science Inner Mongolia University of Science and Technology Baotou Inner Mongolia China; ^3^ School of Automation and Electrical Engineering Inner Mongolia University of Science and Technology Baotou Inner Mongolia China; ^4^ Integrated Research Large Platform for Comprehensive Utilization Technologies of New Important Energy Inner Mongolia University of Science and Technology Baotou Inner Mongolia China; ^5^ Center for Brain Imaging, School of Life Science and Technology Xidian University Xi'an Shaanxi China; ^6^ Engineering Research Center of Molecular and Neuro Imaging Ministry of Education Xi'an Shaanxi China; ^7^ Hainan Free Trade Port Health Medical Research Institute Baoting Hainan China; ^8^ Ganzhou City Key Laboratory of Mental Health the Third People's Hospital of Ganzhou City Ganzhou Jiangxi China

**Keywords:** adolescent health, electroencephalographic, nicotine dependence, sleep architecture disruption, young smokers

## Abstract

Smoking is closely associated with significant disruptions in sleep architecture. Although previous studies have reported altered self‐reported sleep quality in adolescent smokers, relatively few studies using polysomnography have objectively examined critical sleep microstructural components (i.e., slow‐wave activity and sleep spindle characteristics) in young smokers. This study investigated changes in these electroencephalographic indices in adolescent smokers and their associations with smoking‐related characteristics. We recruited 20 young smokers and 16 non‐smokers, collecting their overnight polysomnography data. The Fagerström Test for Nicotine Dependence assessed dependence levels, and the pack‐year index quantified cumulative smoking exposure. We analysed slow waves (0.5–4.5 Hz) in N3 sleep and spindles (11–16 Hz) in N2 sleep, quantifying parameters via the YASA toolbox. Smokers showed impaired N3 slow‐wave integrity (reduced amplitude and slopes) and enhanced N2 spindle activity (increased density, frequency and duration). Slow‐wave amplitude reduction correlated negatively with smoking indices, whereas spindle enhancements correlated positively. Sleep slow waves and spindles may serve as biomarkers for sleep quality in young smokers, deepening understanding of the sleep–smoking relationship.

## Graphical abstract

Compared to non‐smokers, young smokers exhibit impaired N3 sleep slow‐wave integrity and enhanced N2 spindle activity. These microstructural sleep alterations correlate significantly with cigarette exposure severity, offering potential objective biomarkers for early nicotine‐related neural impairment.

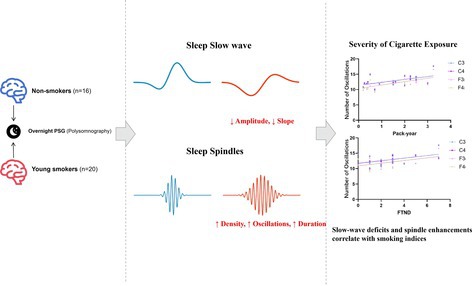

## 1 Introduction

The World Health Organization (WHO) identifies tobacco consumption as the leading risk factor for premature mortality globally, causing approximately 8 million premature deaths annually [1, 2]. Smoking compromises cardiopulmonary and metabolic responses in young, physically active adults during both peak incremental exercise and recovery [3]. Nicotine, the primary alkaloid in tobacco, is the key substance underlying its addictive potential and exerts significant detrimental effects on mental health. Compared to non‐smokers, regular nicotine intake reduces quality of life [4] and sleep quality [5], increases the risk of developing post‐traumatic stress disorder (PTSD) following exposure to traumatic events [6] and elevates the prevalence of depressive and anxiety symptoms [7, 8]. These findings underscore the importance of investigating the impact of smoking on brain development and health outcomes in young smokers.

Sleep, a dynamic and highly active physiological state, is crucial for brain health, cognitive function and metabolic regulation. Its key mechanisms are mediated through distinctive microstructural electroencephalogram (EEG) events [9], among which slow waves during non‐rapid eye movement (NREM) stage 3 (N3) sleep and sleep spindles during NREM stage 2 (N2) sleep are particularly pivotal.

Slow waves (0.5–4.5 Hz), as hallmark oscillations of N3 sleep, reflect the synchronized transitions of cortical neurons between ‘UP’ and ‘DOWN’ states. They serve as core indicators of sleep depth and homeostatic regulation, playing indispensable roles in synaptic plasticity and systems‐level memory consolidation [10, 11].

Concurrently, sleep spindles primarily emerge during N2 sleep [12], typically within a frequency range of 11–16 Hz and with a duration of 0.5–1.5 s [13]. Generated by thalamocortical circuits [14], they are critical for memory processing and exhibit strong associations with intellectual functioning. Interindividual variations in spindle characteristics have been robustly linked to cognitive capacities such as reasoning ability [10].

Though subjective reports (i.e., PSQI) and macrostructural sleep alterations have been extensively documented, such as difficulty initiating sleep, difficulty maintaining sleep and reduced overall sleep quality in smokers [15], they fail to fully capture subtle yet critical changes at the micro level (spindles and slow‐wave morphology). First, regarding sleep microstructure, prior studies predominantly focused on macroscopic power variations or spindle density, whereas in‐depth analysis of detailed slow‐wave morphological characteristics (i.e., amplitude, slope and propagation patterns) and multidimensional spindle features (including oscillatory frequency and duration) remains inadequate. These morphological and temporal‐dynamic properties are considered to more directly reflect the synchronized firing capacity of cortical neuron clusters, propagation efficiency of slow‐wave neural oscillations and intrinsic oscillatory properties of thalamic neurons; consequently, they may serve as more sensitive indicators of nicotine's effects on the brain. Second, adolescence is a critical phase for brain development and sleep pattern maturation, where nicotine exposure may exert unique and long‐lasting impacts on neural circuitry. However, current research has paid relatively scant attention to the sleep microstructure of young smokers. Most crucially, direct quantifiable dose–effect relationships between smoking severity and these specific sleep microstructural traits, along with their potential as sensitive neurobiological markers, have not been systematically investigated. This study aims to explore in depth the relationship between these objective microstructural details of young smokers and smoking status information (Fagerström Test for Nicotine Dependence [FTND] and pack‐years). By focusing on these microstructures, this research can provide more precise biomarkers and mechanistic insights explaining how smoking affects brain function of young smokers during sleep.

## 2 Materials and Methods

### 2.1 Participants

This study enrolled 20 young smokers (mean age of 20.40 ± 1.05 years) and 16 matched non‐smokers (mean age of 19.88 ± 1.03 years), all being undergraduate students at Inner Mongolia University of Science and Technology (IMUST). As members of the same university population, they exhibited high consistency in lifestyle and dietary patterns, including regular sleep–wake cycles, similar academic schedules and typical college dietary habits, which helped reduce interference from external environmental factors on research outcomes. All participants were assessed as right‐handed via the Edinburgh Handedness Inventory [16]. Smokers were diagnosed with nicotine dependence according to the Diagnostic and Statistical Manual of Mental Disorders (DSM‐V); non‐smokers were recruited through concurrently posted advertisements.

Uniform exclusion criteria were applied to both groups: (1) recent use of sedative‐hypnotic medications; (2) severe psychiatric disorders (i.e., major depressive disorder, severe anxiety disorder and schizophrenia) or serious somatic diseases (i.e., acute/chronic heart, liver or kidney failure); (3) neurological conditions potentially altering EEG activity (i.e., Parkinson's disease, Alzheimer's disease and epilepsy); and (4) meeting DSM‐V diagnostic criteria for other substance use disorders. Demographic and smoking characteristics of participants were presented in Table 1.

**TABLE 1:** Demographic and smoking characteristics of smokers and non‐smokers.

Variable	Smokers (mean ± SD)	Non‐smokers (mean ± SD)	*p*
Age (years)	20.4 ± 1.1	20.1 ± 1.3	0.497
Education (years)	14.5 ± 0.9	15.1 ± 1.4	0.3
Sex	Male	Male	
Pack‐year	1.7 ± 0.76		
PSQI	5.2 ± 2.8	3.7 ± 2.1	0.07
SAS	38.1 ± 7.4	43.8 ± 9.2	0.20
SDS	40.8 ± 9.1	45.2 ± 13.6	0.4
ISI	6.5 ± 4.5	4.1 ± 4.0	0.10
SRSS	19.3 ± 4.5	17.1 ± 4.4	0.15
FTND	2.7 ± 1.9		

*Note:* The comparisons between groups were conducted using independent samples *t*‐test. There were no significant differences in age or education level observed between young smokers and non‐smokers. All demographic and scale measures were obtained at baseline. The pack‐year index was calculated by multiplying the number of packs of cigarettes smoked per day by the number of years the person has smoked.

### 2.2 Procedures

Prior to polysomnography (PSG) recording, all participants completed standardized scale assessments including the Pittsburgh Sleep Quality Index (PSQI), Self‐Rating Depression Scale (SDS), Self‐Rating Anxiety Scale (SAS), Insomnia Severity Index (ISI) and Self‐Rating Scale of Sleep (SRSS) to comprehensively evaluate sleep quality, anxiety and depression levels [17]. For smokers, the FTND was additionally administered to assess nicotine dependence severity, whereas cumulative nicotine exposure was quantified using pack‐year index [18]. All subjects underwent overnight PSG monitoring. This study was approved by the Ethics Committee of the First Affiliated Hospital of Baotou Medical College, of IMUST (No. ChiCTR2100042449), with written informed consent obtained from all participants. No smoking cessation interventions, pharmacological treatments or other interventions were implemented during the study period. Furthermore, to minimize the influence of exogenous substances on sleep architecture, all participants were instructed to strictly abstain from caffeine, alcohol and any non‐prescribed medications for at least 24 h prior to the PSG recording.

### 2.3 Polysomnography

All participants underwent supervised overnight PSG monitoring in the laboratory environment from 22:00 to 06:00. Lights were turned off at 22:00 sharp, with subjects required to remain in bed attempting to sleep, and were awakened at 06:00 am the next day.

PSG data were acquired using a SOMNOscreen plus EEG/polygraphic system (SOMNOmedics GmbH, Germany). Electrode placement followed the international 10‐20 system. The montage included EEG electrodes (F3, F4, C3, C4, O1, O2, with M1 and M2 as references, and a ground electrode), electrooculography electrodes, electrocardiography electrodes and respiratory inductive plethysmography belts.

### 2.4 EEG Data Preprocessing

This study selected partial recording leads for sleep data analysis, including frontal (F3 and F4) and central (C3 and C4) electrodes. These specific positions were chosen because the frontal region is the predominant generator of sleep slow waves, whereas sleep spindles exhibit maximal amplitude and density over the central and frontal cortical areas, making them the most representative topographical sites for evaluating these microstructural features. Raw data were referenced to the vertex (Cz) electrode during acquisition and offline re‐referenced to averaged mastoid reference. Overnight sleep data were segmented into 30‐s epochs according to the American Academy of Sleep Medicine standards, with sleep staging manually performed by specialists [19].

N2 and N3 sleep stages were selected for EEG analysis because they represent core components of NREM sleep. Specifically, N2 demonstrates high stability and constitutes the largest proportion of sleep, exhibiting the most prominent spindle activity, whereas N3, as deep sleep, shows the most significant slow‐wave activity. Together, they reflect key features of sleep microstructure [20].

EEG processing utilized MNE‐Python toolkit [21]: Raw data were down‐sampled to 512 Hz after import, followed by 0.1–40 Hz band‐pass filtering; Independent component analysis using Fast‐ICA algorithm was applied, with components visually screened by experienced researchers to remove electromyographic (EMG) and ocular artefact‐related components. Artefact‐free EEG signals were reconstructed from retained components; identified artefacts underwent manual detection and removal [22]; epochs with excessive noise/artefacts were excluded by final visual inspection. All EEG electrodes' impedances were maintained below 10 kΩ.

### 2.5 EEG Data Analyses

All sleep event detection was performed using the YASA (Yet Another Spindle Algorithm, v0.6.5) library in Python (v3.9). The core methodology of YASA has been detailed in publications by Vallat and Walker [23].

#### 2.5.1 Slow‐Wave Analyses

Slow waves were automatically detected on C3, C4, F3 and F4 electrodes using the *yasa.sw_detect* function, which was restricted to N3 sleep by setting. The sampling frequency of preprocessed data was 512 Hz.

Detection parameters were set based on typical physiological characteristics of slow waves and YASA's recommended defaults to accurately identify their frequency, duration and amplitude attributes: frequency range: 0.3–1.5 Hz; negative half‐wave duration: 0.3–1.5 s; positive half‐wave duration: 0.1–1.0 s; amplitude thresholds: negative peak amplitude: 40–200 μV, positive peak amplitude: 10–150 μV, peak‐to‐peak amplitude: 75–350 μV. For z‐score normalized data, thresholds adjusted to negative peak amplitude ≥ 1 standard deviation (SD), positive peak amplitude ≥ 1 SD and peak‐to‐peak amplitude between 3 and 10 SD.

For each detected slow wave, the following features were extracted: time to negative peak, time to zero‐crossing (negative‐to‐positive), time to positive peak, duration, negative peak amplitude, positive peak amplitude, peak‐to‐peak amplitude (the voltage difference between the maximum positive and minimum negative peaks), phase–amplitude coupling within a 2‐s window centred at the negative peak, slope (negative peak to zero‐crossing) and frequency.

#### 2.5.2 Spindle Analyses

Sleep spindles were automatically detected on C3, C4, F3 and F4 electrodes using *yasa.spindles_detect* function, which was restricted to N2 sleep. The sampling frequency of preprocessed data was 512 Hz.

Spindle detection employed multi‐threshold criteria to enhance robustness and specificity in distinguishing true spindles from noise/non‐specific oscillations: spindle frequency range: 12–16 Hz; broadband frequency range: 1–30 Hz (for relative power calculation); minimum and maximum duration: 0.5–2 s. If two spindles occurred < 500 ms apart, they were merged. Detection threshold dictionary included relative power, moving correlation between raw and sigma‐filtered signals and moving root mean square (RMS) of sigma‐filtered signals. Mandatory multi‐channel co‐occurrence was not required.

For each detected sleep spindle, the following features were extracted: mean duration, spindle density, detrended peak‐to‐peak amplitude, relative power (the ratio of spindle‐band power to total broadband power), median absolute power in the 12–16 Hz band computed via the Hilbert transform, RMS amplitude, instantaneous frequency, number of oscillations (defined by the positive peak count) and symmetry index (the normalized position of the most prominent peak, ranging from 0 to 1, where 0.5 indicates perfect symmetry).

### 2.6 Statistical Analysis

Statistical analyses first assessed data normality using Shapiro–Wilk tests. Based on the normality outcomes, Welch *t*‐tests (for normally distributed data) or Mann–Whitney *U* tests (for non‐normally distributed data) were applied to analyse group differences in slow‐wave morphological features and sleep spindle characteristics between young smokers (*n* = 20) and non‐smokers (*n* = 16). For multiple comparisons, the false discovery rate correction was used to control false‐positive risks, with the significance threshold set at α = 0.05. The associations between slow‐wave characteristics during N3 sleep, spindle characteristics during N2 sleep and smoking‐related variables were analysed using Pearson correlation coefficients (for normally distributed data) or Spearman correlation coefficients (for non‐normally distributed data), with a statistical significance threshold set at *p* < 0.05. Furthermore, to evaluate the adequacy of our sample size (*n* = 36), a post hoc sensitivity power analysis was conducted using G*Power software (Version 3.1). Given the total sample size of 36 participants, an alpha level of 0.05, and a statistical power of 80% (1 − β = 0.80), the minimum detectable effect size (Cohen's *d*) was approximately 0.96. Although our study successfully detected significant between‐group differences with moderate effect sizes (e.g., Hedges' *g* ranging from 0.41 to 0.47 for specific slow‐wave and spindle parameters), the relatively modest sample size indicates that the study may be underpowered to detect smaller physiological effects. Therefore, these findings should be considered exploratory.

## 3 Results

### 3.1 Demographics and Scores on the Subjective Scale

Table 1 lists demographic variables and scale scores. Young smokers and non‐smokers showed no significant differences in age or educational attainment. Regarding subjective scale scores, although young smokers exhibited a downward trend in PSQI scores (*p* = 0.07), no significant differences were observed between young smokers and non‐smokers across all scale scores, including sleep state assessments and anxiety–depression scores.

### 3.2 Slow Wave

**FIGURE 1:**
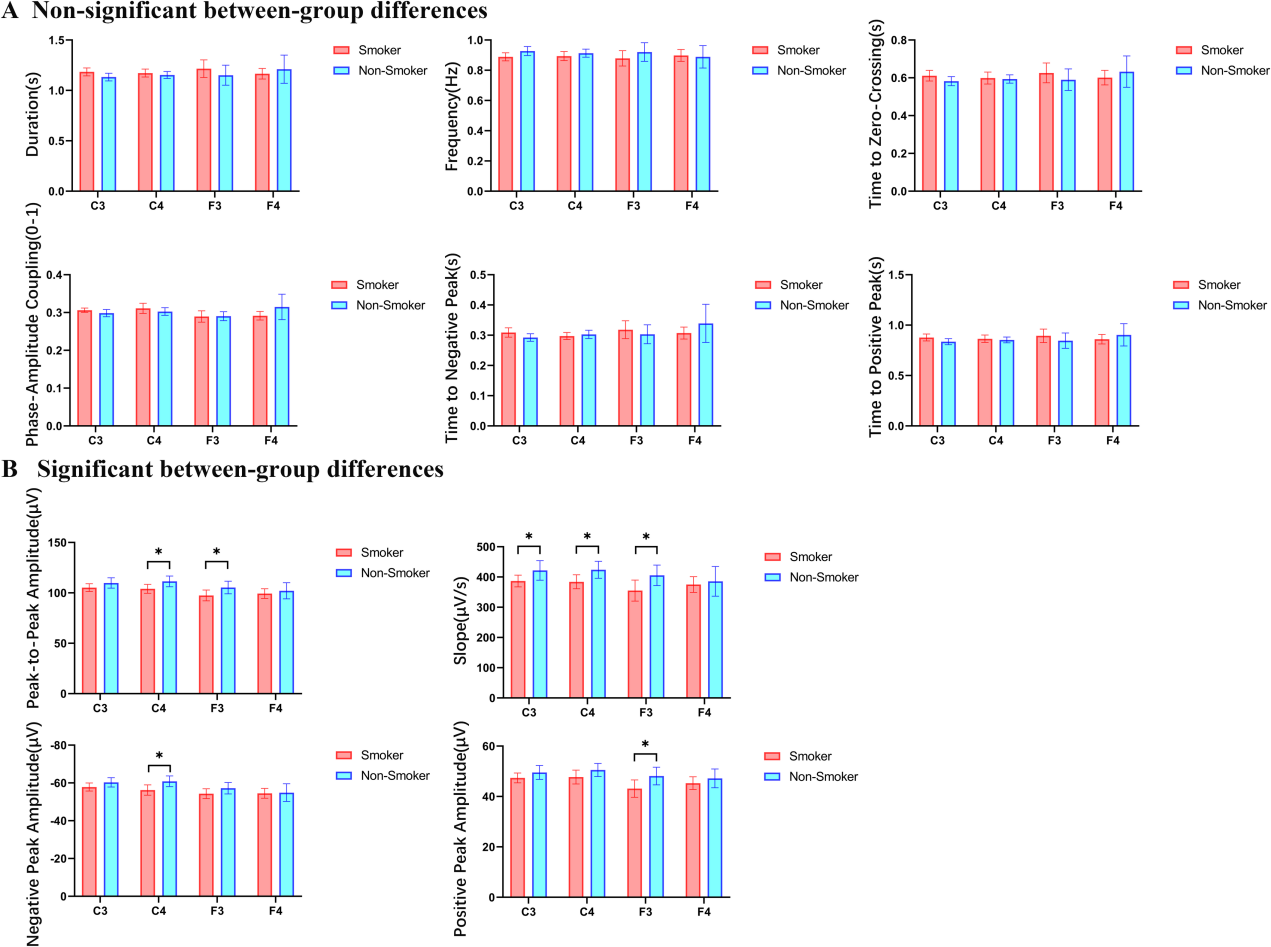
Comparison of slow‐wave characteristics during N3 sleep between young smokers and non‐smokers. (A) Slow‐wave parameters showing no significant between‐group differences. (B) Slow‐wave parameters showing significant between‐group differences. Young smokers exhibited significant alterations in specific slow‐wave metrics, including reduced peak‐to‐peak amplitude, decreased decay slope and altered negative/positive peak amplitudes compared to non‐smokers. Bar charts illustrate group means ± standard error of the mean (SEM). *Note:* * indicates FDR‐adjusted *p* < 0.05, ** indicates FDR‐adjusted *p* < 0.01. C3/C4, left/right central electrodes; F3/F4, left/right frontal electrodes.

Analysis of N3 sleep slow‐wave parameters revealed significant between‐group differences between young smokers and non‐smokers at electrodes C3, C4 and F3, with parameter‐specific variation in these differences (Figure 1).

No statistically significant differences were observed between groups for time to negative peak, time to zero‐crossing, time to positive peak, phase–amplitude coupling or frequency (*p* > 0.05) (Figure 1A).

In contrast, several parameters showed significant group differences (Figure 1B). Specifically, peak‐to‐peak amplitude was reduced at C4 (*t* = −2.2747, *p* = 0.0293) and at F3 (U = 95.00, Hedges' *g* = 0.41, *p* = 0.04); slopes were significantly diminished at C3 (*t* = −2.0527, *p* = 0.0479), C4 (*t* = −2.3300, *p* = 0.0259) and F3 (*t* = −2.1565, *p* = 0.0382). Additionally, negative peak amplitude was increased at C4 (*t* = 2.4468, *p* = 0.0197), whereas positive peak amplitude was decreased at F3 (U = 91.00, Hedges' *g* = 0.44, *p* = 0.0292).

In summary, young smokers exhibited abnormalities in N3 sleep slow‐wave parameters at C3, C4 and F3 electrodes, with no significant differences in other slow‐wave characteristics.

### 3.3 Spindle

**FIGURE 2:**
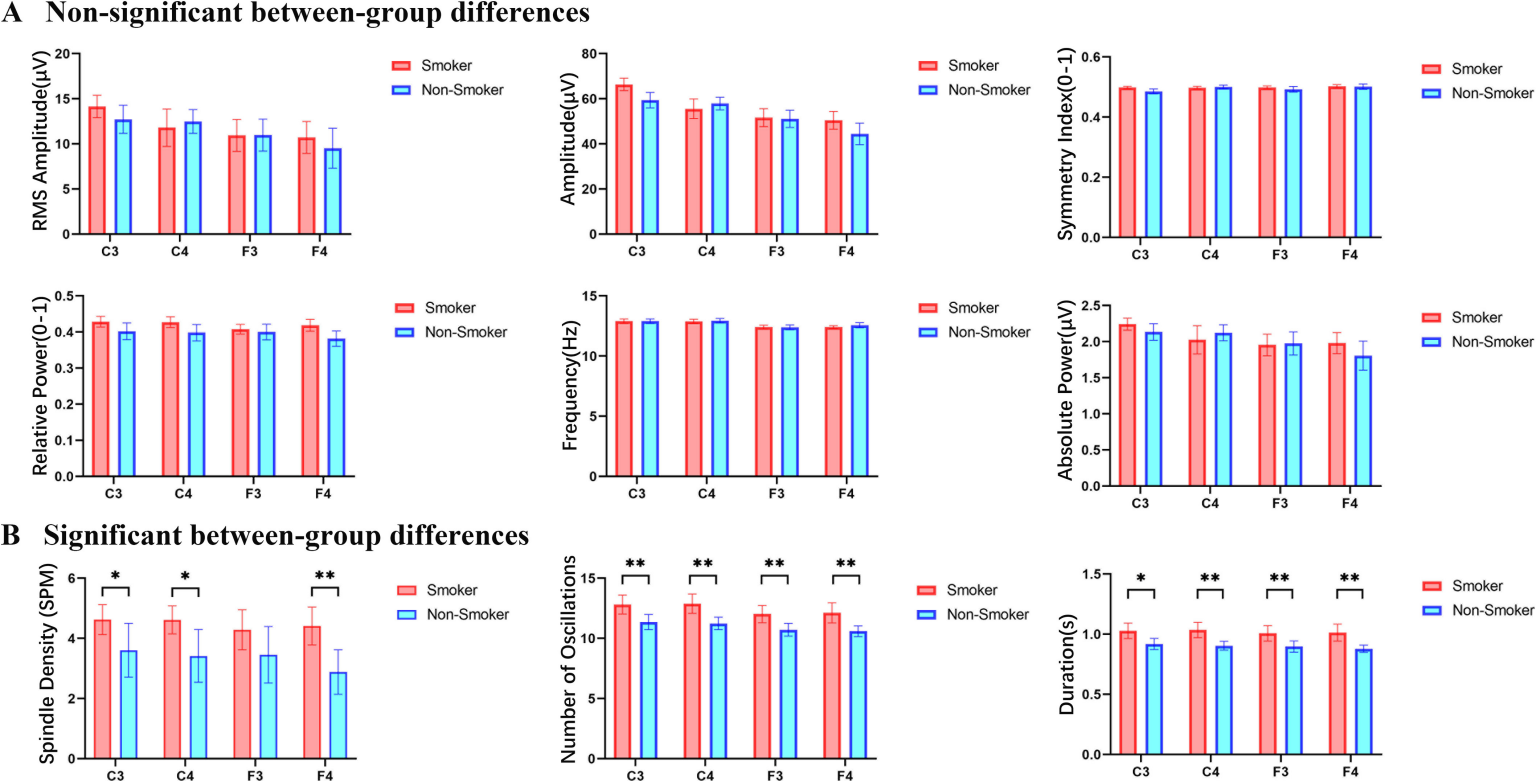
Comparison of spindle characteristics and parameter analysis during N2 sleep between young smokers and non‐smokers. (A) Spindle parameters showing no significant between‐group difference. (B) Spindle parameters showing significant between‐group differences. Young smokers exhibited significantly enhanced spindle activity, characterized by increased spindle density, number of oscillations and duration across the analysed cortical regions. Bar charts illustrate group means ± standard error of the mean (SEM). *Note:* * indicates FDR‐adjusted *p* < 0.05, ** indicates FDR‐adjusted *p* < 0.01. C3/C4, left/right central electrodes; F3/F4, left/right frontal electrodes.

Analysis of N2 sleep spindle parameters revealed significant between‐group differences between young smokers and non‐smokers at electrodes C3, C4, F3 and F4, with parameter‐specific variation in these differences (Figure 2).

No statistically significant between‐group differences were observed for spindle RMS amplitude, amplitude, symmetry index, relative power frequency or absolute power (*p* > 0.05) (Figure 2A).

In contrast, several parameters exhibited significant group differences. Specifically, spindle density was significantly increased at C3 (*t* = 2.2179, *p* = 0.0333), C4 (*t* = 2.5626, *p* = 0.0173) and F4 (*t* = 3.3405, *p* < 0.01). Oscillation was significantly increased at C3 (*t* = 2.234, *p* < 0.01), C4 (*t* = 3.4101, *p* < 0.01), F3 (*t* = 2.9372, *p* < 0.01) and F4 (*t* = 3.247, *p* < 0.01). Furthermore, duration was significantly prolonged at C3 (U = 235.0000, Hedges' *g* = 0.42, *p* = 0.0177), C4 (*t* = 3.4966, *p* < 0.01), F3 (U = 251.0000, Hedges' *g* = 0.45, *p* < 0.01) and F4 (U = 260.0000, Hedges' *g* = 0.47, *p* < 0.01) (Figure 2B).

In summary, young smokers exhibited increased spindle density at C3, C4 and F4, alongside elevated oscillation frequency and prolonged duration across all analysed electrodes (C3, C4, F3 and F4) during N2 sleep, with no significant differences observed in other spindle characteristics.

### 3.4 Association Between Sleep EEG Features and Smoking Status

**FIGURE 3:**
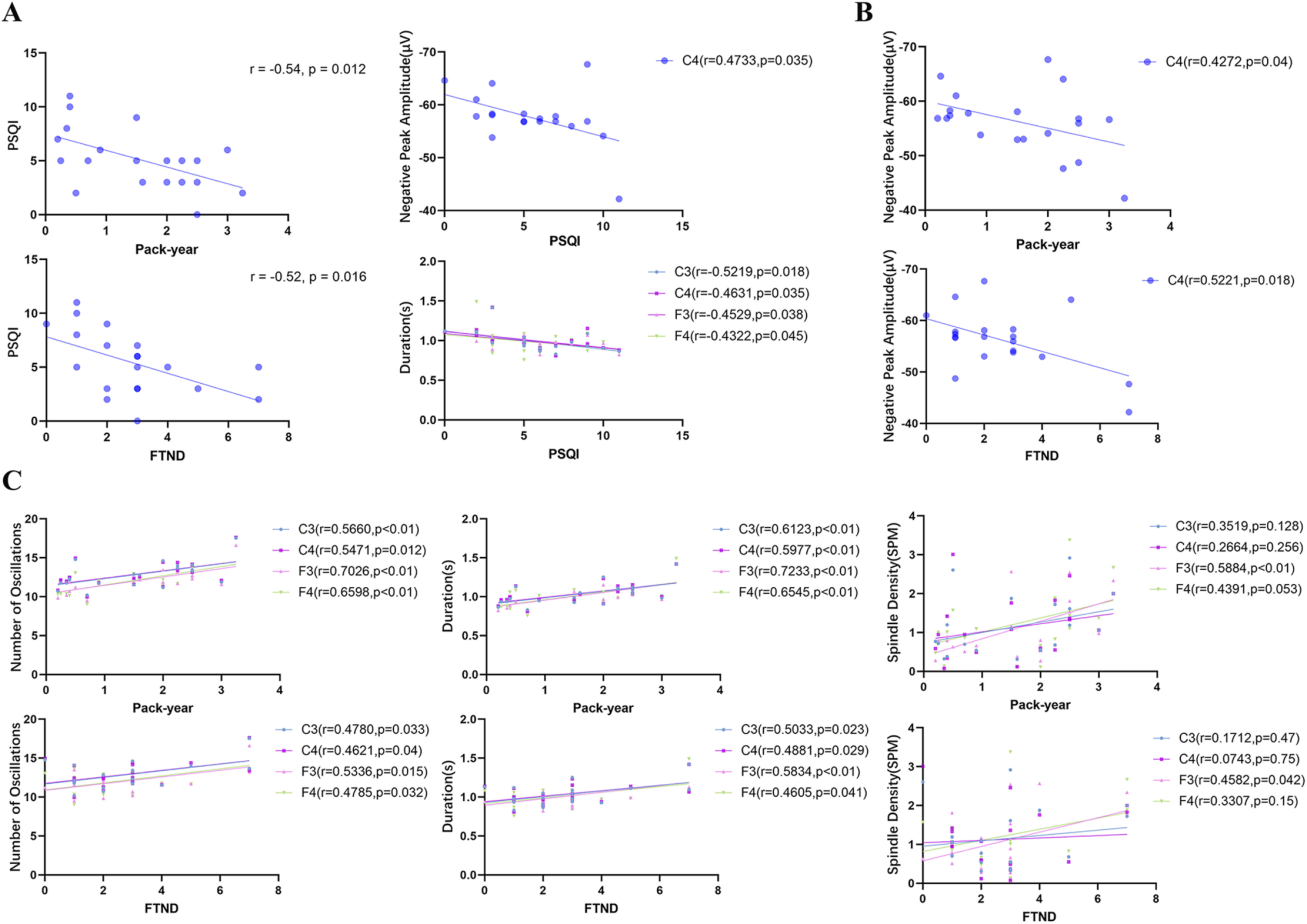
Correlations between sleep EEG microstructural parameters, subjective sleep quality, and smoking characteristics in young smokers. Scatter plots illustrating the linear relationships between specific variables. The solid lines represent the linear regression fits. Pearson's or Spearman's correlation coefficients (*r*) and corresponding raw *p*‐values are presented in each panel. (A) Correlations of subjective sleep quality (PSQI scores) with smoking indices (pack‐years and FTND) and specific EEG parameters (negative peak amplitude and duration). (B) Correlations of N3 sleep slow‐wave characteristic (negative peak amplitude) with smoking indices (pack‐years and FTND). (C) Correlations of N2 sleep spindle characteristics (number of oscillations, duration, and spindle density) with smoking indices (pack‐years and FTND) across different electrode sites. FTND, Fagerström Test for Nicotine Dependence; PSQI, Pittsburgh Sleep Quality Index. C3/C4, left/right central electrodes; F3/F4, left/right frontal electrodes.

First, we examined the link between sleep quality (assessed by PSQI) and smoking exposure indicators. PSQI was negatively correlated with pack‐year (*r* = −0.54, *p* = 0.032) and FTND (*r* = −0.52, *p* = 0.036), indicating that greater smoking exposure was associated with poorer sleep quality (higher PSQI scores). Additionally, PSQI showed negative correlations with slow‐wave negative peak amplitude and spindle duration (for *r*/*p* values, see Figure 3A).

Subsequently, correlation analyses were performed between significantly altered slow‐wave/spindle features and pack‐year/FTND scores across smoking statuses. Results revealed that slow‐wave negative peak amplitude negatively correlated with both pack‐year and FTND (for *r*/*p* values, see Figure 3B). For spindles, F3 spindle density positively correlated with pack‐year and FTND; number of oscillations at all electrodes positively correlated with pack‐year and FTND; duration at all electrodes positively correlated with pack‐year and FTND (for *r*/*p* values, see Figure 3C).

## 4 Discussion

This study aimed to comprehensively investigate specific alterations in sleep microstructure among young smokers, with particular focus on slow‐wave morphological characteristics during N3 sleep and multidimensional spindle features during N2 sleep. We further explored the associations between these changes and smoking pack‐years, as well as FTND. Key findings confirmed our hypotheses: Compared with non‐smoking peers, young smokers exhibited systematic alterations in both slow‐wave intensity during N3 sleep and spindle activity intensity during N2 sleep. Critically, the magnitude of changes in several altered parameters, including slow‐wave negative peak amplitude and spindle characteristics (number of oscillations/duration across all electrodes, spindle density in specific electrodes), showed significant correlations with pack‐years and FTND. This suggests that these sleep EEG features may serve as sensitive biomarkers of smoking‐related neurobiological alterations.

Nicotine exerts fundamental effects on neural circuits through evolutionarily conserved neurochemical mechanisms across mammalian systems: It binds to nicotinic acetylcholine receptors (nAChRs), especially α4β2 subtypes highly expressed in the thalamus and cortex, to enhance neuronal excitability [24, 25]. Concurrently, it disrupts the balance between inhibitory GABAergic and excitatory glutamatergic transmission: Chronic exposure typically reduces GABAergic inhibition (via desensitization of inhibitory inputs) while promoting glutamate release (via α7‐nAChR stimulation on presynaptic terminals), shifting the excitation–inhibition balance towards overactivation [26, 27]. These shared neurochemical changes form the basis for nicotine‐induced alterations in both slow‐wave and spindle activity, consistent with findings that transdermal nicotine administration also modulates EEG spectral properties during sleep in healthy adults [28].

For slow waves, which are generated by synchronized cortical UP/DOWN state transitions coordinated via thalamocortical circuits [29, 30] nicotine's excitation‐biased imbalance directly impairs the neural coordination required for high‐amplitude, steep oscillations. The heightened cortical excitability (from reduced GABA inhibition and increased glutamate) disrupts pyramidal neurons' ability to sustain widespread, synchronous DOWN states [31, 32], manifesting as reduced amplitude and slope in specific regions (C4 and F3). Additionally, nicotine‐induced activation of the mesolimbic dopaminergic system (via VTA–NAc projections) elevates cortico‐subcortical arousal, antagonizing the hyperpolarized state necessary for deep N3 sleep and further suppressing slow‐wave generation.

For sleep spindles, which are generated by oscillatory interactions between the thalamic reticular nucleus (TRN) and dorsal thalamic nuclei [33], nicotine's effects converge on enhancing TRN excitability. Activation of TRN nAChRs potentiates T‐type calcium electrodes critical for burst firing [34, 35], increasing spindle density and duration. Concurrently, chronic nicotine‐induced desensitization of GABAergic inputs to TRN induces a disinhibited state, lowering the threshold for sustained oscillatory bursts [27, 35]. This TRN hyperactivity, combined with globally heightened neuronal excitability, explains the elevated oscillatory frequency and prolonged duration of spindles across all electrodes, reflecting altered thalamic intrinsic oscillatory properties [36].

Notably, whereas both phenomena arise from nicotine's disruption of excitation–inhibition balance, their circuit‐specific effects differ: Slow‐wave attenuation reflects impaired cortical synchrony and heightened arousal, whereas spindle enhancement reflects hyperactive thalamic oscillatory generation, this divergence underscores the multifaceted impact of nicotine on sleep microarchitecture.

Regarding the observed spindle enhancement, it remains debated whether this represents a purely pathological thalamocortical over‐excitation or a distinct compensatory neuroadaptive mechanism. On one hand, chronic nicotine exposure induces persistent hyper‐excitability in thalamic circuits, pushing the network into a state of forced oscillatory bursting [34, 35]. On the other hand, sleep spindles are widely recognized for their sleep‐protective role, gating sensory processing and preventing environmental arousals. Given the disruptive, arousal‐promoting effects of nicotine on the cortex, the global upregulation of spindle density and duration might reflect a compensatory effort by the brain to maintain sleep continuity and protect against nicotine‐induced sleep fragmentation. Future functional neuroimaging studies are necessary to disentangle these underlying neural dynamics.

Although our study did not directly assess cognitive and emotional outcomes, these specific EEG microstructural alterations serve as objective biomarkers with significant functional implications for young smokers. Slow‐wave activity is fundamentally linked to homeostatic sleep drive, synaptic downscaling and memory consolidation [10, 11]. The observed attenuation of slow‐wave amplitude and slope suggests an impairment in these critical restorative processes. Interestingly, this progressive decline in slow‐wave integrity closely mirrors the neurophysiological changes typically observed in normal ageing, suggesting that early‐stage smoking might provoke neurofunctional decline akin to accelerated brain ageing. Conversely, the enhancement of sleep spindles—structures vital for motor learning and memory integration—further reflects this complex neuroadaptive shift. Behaviourally, these microstructural disruptions could underlie the daytime cognitive deficits, impaired emotional regulation and heightened vulnerability to anxiety and depression frequently reported in adolescent smokers [7, 8]. Furthermore, altered spindle dynamics might play a subtle role in the consolidation and maintenance of nicotine dependence itself, highlighting a bidirectional relationship between sleep architecture and addiction circuitry.

Recognizing these specific oscillatory abnormalities opens new avenues for circuit‐targeted therapeutics. Indeed, recent advances in non‐invasive neuromodulation, such as personalized transcranial alternating current stimulation (tACS) and transcranial temporal interference stimulation (tTIS), have successfully reduced craving in other substance use disorders by precisely modulating neural oscillations and specific brain circuits [37, 38]. Future interventions could potentially utilize similar techniques to target and normalize these sleep‐related EEG biomarkers, thereby offering a novel therapeutic strategy to alleviate nicotine dependence and facilitate smoking cessation.

### 4.1 Limitations

This study has several limitations. First, the sample comprised exclusively young male undergraduates. This limitation arose primarily from logistical and staffing constraints associated with the overnight application of PSG electrodes and monitoring. Nonetheless, restricting the sample to males in this initial exploratory study inherently served to eliminate the potential confounding effects of female hormonal fluctuations (e.g., menstrual cycle phases) on sleep architecture and body temperature, ensuring a more homogeneous baseline. Second, as indicated by our power analysis, the relatively small sample size limits statistical power, meaning we may have been underpowered to detect smaller physiological effects. Third, the cross‐sectional design precludes establishing definitive causal relationships; despite strong correlations observed, smoking cannot be conclusively proven to directly cause these alterations. Fourth, smoking metrics (pack‐years, FTND) relied on self‐reporting, and the absence of objective biological markers, such as exhaled carbon monoxide (CO) or cotinine levels, may introduce assessment bias. Fifth, we did not strictly control for all potential confounding factors that might influence sleep, such as daily stress levels or undetected subclinical respiratory conditions. Finally, the study did not directly assess associations between sleep EEG changes and cognitive functions. Future longitudinal investigations are essential to establish causality and clarify the detailed neural mechanisms. Specifically, tracking microstructural alterations over time will help clarify how these EEG markers evolve during smoking initiation, maintenance and cessation, particularly given the evidence that sleep parameters may normalize after quitting [39].

## 5 Conclusions

Theoretically, this study provides the first systematic report of specific alterations in multidimensional slow‐wave and spindle characteristics among young smokers, revealing dose‐dependent relationships. These findings deepen our understanding of how early‐stage smoking impacts critical neural oscillatory patterns.

Identified slow‐wave and spindle parameters and their correlations with smoking metrics demonstrate potential as quantifiable, objective biomarkers for assessing severity of smoking‐related neural impairment, predicting individual risk trajectories and monitoring therapeutic efficacy (i.e., smoking cessation interventions). Furthermore, elucidating nicotine's disruption of sleep oscillatory dynamics, especially slow wave‐spindle coupling critical for memory consolidation, establishes a neurophysiological foundation for explaining smoking‐associated cognitive changes.

## Author Contributions

**Shuailin Ding:** writing – original draft, validation, methodology, formal analysis, data curation, conceptualization. **Ting Xue:** writing – review and editing, funding acquisition, formal analysis, data curation, conceptualization. **Fang Dong:** writing – review and editing, funding acquisition. **Yongxin Cheng:** methodology, formal analysis. **Yuxin Ma:** resources, investigation. **Juan Wang:** data curation, visualization, software. **Dahua Yu:** writing – review and editing, funding acquisition, formal analysis, data curation, conceptualization. **Kai Yuan:** writing – review and editing.

## Funding

This work was supported by Chinese National Programs for Brain Science and Brain‐like Intelligence Technology (2022ZD0214500); the STI2030‐Major Project (2022ZD0207100); the National Natural Science Foundation of China (82260359, 82371500, U22A20303, 61971451); the Natural Science Foundation of Inner Mongolia (2023QN08007, 2025MS08027, 2025MS08098); the Fundamental Research Funds for the Universities of Inner Mongolia (2023QNJS204, 2023QNJS206, 2024QNJS119); and the Development Program for Young Talents of Science and Technology in Universities of Inner Mongolia (NJYT24030).

## Ethics Statement

The current study complied with the Declaration of Helsinki and got permission from the Medical Ethical Committee of the First Affiliated Hospital of Baotou Medical College, Inner Mongolia University of Science and Technology. The study was registered in the Chinese Clinical Trial Registry (No. ChiCTR2100042449). Prior to the experiment, each subject signed an informed consent form after fully understanding the procedures and precautions.

## Conflicts of Interest

The authors declare no conflicts of interest.

## Data Availability

The datasets used and analysed during the current study are available from the corresponding author upon reasonable request.
